# The Role of microRNAs in Bovine Infection and Immunity

**DOI:** 10.3389/fimmu.2014.00611

**Published:** 2014-11-27

**Authors:** Nathan Lawless, Peter Vegh, Cliona O’Farrelly, David J. Lynn

**Affiliations:** ^1^Animal and Bioscience Research Department, Animal and Grassland Research and Innovation Centre, Teagasc, Dunsany, Meath, Ireland; ^2^School of Biochemistry and Immunology, Trinity College Dublin, Dublin, Ireland; ^3^School of Genetics and Microbiology, Smurfit Institute of Genetics, Trinity College Dublin, Dublin, Ireland; ^4^South Australian Health and Medical Research Institute, North Terrace, Adelaide, SA, Australia; ^5^School of Medicine, Flinders University, Bedford Park, SA, Australia

**Keywords:** bovine, *Bos taurus*, microRNA, miRNA, immune system

## Abstract

MicroRNAs (miRNAs) are a class of small, non-coding RNAs that are recognized as critical regulators of immune gene expression during infection. Many immunologically significant human miRNAs have been found to be conserved in agriculturally important species, including cattle. Discovering how bovine miRNAs mediate the immune defense during infection is critical to understanding the etiology of the most prevalent bovine diseases. Here, we review current knowledge of miRNAs in the bovine genome, and discuss the advances in understanding of miRNAs as regulators of immune cell function, and bovine immune response activation, regulation, and resolution. Finally, we consider the future perspectives on miRNAs in bovine viral disease, their role as potential biomarkers and in therapy.

## Introduction

MicroRNAs (miRNAs) are short, non-coding RNAs, which post-transcriptionally regulate gene expression (1). Since their initial discovery in 1993 (2), studies have convincingly demonstrated critical roles for miRNAs in the regulation of many cellular processes, such as differentiation and proliferation (3). There is also substantial evidence, primarily from human and mouse studies, which miRNAs regulate innate and adaptive immune mechanisms (4, 5). However, the regulatory potential of miRNAs in agriculturally important species, such as cattle, is poorly explored. Here, we aim to review the role of miRNAs in bovine immune and inflammatory systems.

## MicroRNA Biogenesis

MicroRNAs are transcribed by RNA Polymerase II or III as primary miRNAs (pri-miRNAs) in the nucleus, and are then processed into pre-miRNAs by the Microprocessor multiprotein complex, and the co-factor DiGeorge Syndrome Critical Region 8 (DGCR8/Pasha) (6). Following export to the cytoplasm by exportin 5 (XPO5), a mature 22 nucleotide long duplex is formed by an RNAse type III enzyme, called Dicer (7). The duplex, together with Trans-Activation Responsive RNA-Binding Protein 2 (TARBP2) and Argonaute (AGO) family proteins, form a complex, which triggers the assembly of the RNA-Induced Silencing Complex (RISC). One miRNA strand is removed, and the other strand guides RISC to its target mRNA via base-pairing (6). The recognition of the target binding site, which can be found in the coding or the untranslated regions (UTR) of the mRNA, mostly depends on a part called the seed sequence (nucleotides 2–7 at the 5′ end) of the miRNA (7). Although both strands of the duplex can be functional, usually only one strand is used (8).

MicroRNAs are evolutionarily conserved and have been found in all eukaryotes from unicellular species to mammals (9). To date, 2588 have been identified in the human genome, 1915 in mouse, and 793 in bovine (miRBase version 21, http://www.mirbase.org) (10). Originally, miRNAs were thought to mainly regulate gene expression by inhibiting translation, however, transcriptional regulation has been shown recently to be the primary mechanism used by miRNAs to influence gene expression in mammals (11). Several important factors determine how miRNAs function, including the location and number of target sites within mRNA 3′ UTRs (or other gene regions), RNA-binding proteins (RBPs), which interfere with RISC binding, RISC co-factors, and the modification of RISC-components (12). Transcripts with target sites for a common miRNA compete for recognition. These competing endogenous RNAs (ceRNA), such as other mRNAs, long non-coding RNAs (lncRNA), pseudogene transcripts, and independently transcribed UTRs, can reduce the effect of specific miRNAs *in vivo*. Binding to target sites also protects miRNAs from degradation, in a process called target mediated miRNA protection (TMMP) (12). Individual miRNAs can have many targets, explaining how relatively small numbers of alternatively expressed miRNAs can have a large impact on gene regulation and control several diverse biological processes.

## MicroRNAs in Immunity and Infection

Cell-type specific expression of miRNAs has been demonstrated in innate and adaptive immune cells (13, 14) and there is a growing body of evidence that miRNAs regulate the differentiation, development, and function of these cells (15–17). Hematopoietic stem cell differentiation into myeloid and lymphoid lineages, for example, has been shown to be under the influence of several miRNAs, including miR-125b, miR-126, and miR-196b (4, 18). Additionally, the deletion of the *dicer1* gene, which is critical for proper miRNA processing, results in impaired T cell development (19), while miR-17–92, miR-150, and miR-155 have been demonstrated to be critical for B cell development. Other roles for miRNAs in regulating adaptive immunity have also been shown, including the regulation of B and T lymphocyte functions, including antibody production, by miR-155 (20–22). Activation of the innate immune system is also regulated by miRNAs (23). The human miRNA, miR-146a, for example, has been shown to target tumor necrosis factor receptor-associated factor 6 (TRAF6) and interleukin-1 (IL1) receptor-associated kinase (IRAK1), key regulatory nodes, which control innate immune signaling in response to lipopolysaccharide (LPS) (24). Similarly, miR-19a has been shown to regulate expression of SOCS 3, an important suppressor of cytokine signaling (25).

MicroRNAs have also been clearly demonstrated to have important roles in regulating responses to infection (26). In particular, several miRNAs have been identified to have important functions in regulating immune responses to mycobacterial infection (27). Tumor necrosis factor (TNF) biosynthesis, for example, is inhibited by *Mycobacterium tuberculosis* – an intracellular mycobacterial pathogen that infects alveolar macrophages – by altering levels of human macrophage miRNAs, including miR-125b and miR-155, for its own benefit (28). Similarly, miR-29 and miR-99b regulate the production of multiple cytokines, including IFN-γ and TNF-α, which control *M. tuberculosis* growth (29, 30). miRNAs are frequently evolutionarily conserved and many of these miRNAs have orthologs in cattle, therefore data from human and mouse studies can provide a roadmap for revealing miRNAs likely to have important roles in bovine infectious diseases. Many miRNAs, however, exhibit pathogen or stimulus-specific response profiles and certain families of miRNAs are expanded or contracted in the bovine genome.

## MicroRNAs in the Bovine Genome

The first studies demonstrating miRNA expression in bovine tissues were undertaken in 2007 (31, 32). Since then, 793 mature miRNAs, encoded on all 30 chromosomes, have been identified in the *Bos taurus* genome. These miRNAs account for approximately a quarter of all the 3825 non-coding RNAs predicted in the genome by Ensembl (v75) (33). Typically, miRNAs have been grouped into families based on shared sequence similarity of the miRNA seed region (2–8 nt), the mature sequence, or the precursor miRNA sequence (34). Often, miRNA families can be found clustered with target genes in specific genomic regions (35). Many human miRNAs, including some of the most extensively studied immune-related miRNAs, share significant functional and sequence similarities to their bovine counterparts indicating evolutionary conservation and, putatively, conservation of function. The human miRNA, hsa-miR-155, for example, is a perfect homolog to its bovine counterpart bta-miR-155. In humans, this miRNA acts as an anti-inflammatory agent targeting the Toll-like receptor/Interleukin-1 receptor (TLR/IL1R) inflammatory pathway (36). Another miRNA with a conserved bovine ortholog, hsa-miR-146a-5p, is known to negatively regulate the retinoic acid-inducible gene 1 (RIG-I) pathway in humans by suppressing TRAF6 and IRAK1 during viral infection (37). There is also an exact seed sequence match between hsa-miR-146a-5p, bta-miR-146a, and mmu-miR-146a-5p.

While there is significant conservation of miRNAs between species, there are also notable differences that very likely have functional consequences. There are numerous cases, for example, of miRNAs found in the human genome that are apparently absent in bovine. Some of these differences may be due to better annotation of the human microRNAome but clearly there are real differences too. The human miRNA, hsa-miR-198, for example, has a role in human immunity and has no apparent homolog in the bovine genome. This miRNA targets the Cyclin T1 gene (CCNT1), which acts as a co-factor for HIV-1 (38).

In addition to single miRNA differences in the repertoire of human and bovine miRNAs, there are also several cases where entire families or clusters of miRNAs that are present in human have yet to be discovered or do not exist in the bovine genome. These include the majority of miRNAs numbered from miR-550 to miR-640; some 200 miRNAs, which include the hsa-miR-515 cluster (11 miRNAs), and interestingly, the miR-548 family. The miR-548 family comprises of over 70 miRNAs whose expression to date has only been described in simians. Members of this miRNA family have been shown to target interferon-λ1 (IFN-λ1), modulating the primate interferon response to viral infections (39).

There are also several miRNA families in the bovine genome that are apparently species-specific, at least when compared to available genomes. The bta-miR-2284 and bta-miR-2285 families, for example, encode more than 100 mature miRNAs in the bovine genome but do not appear to have homologs in either human or mouse. These miRNA families have been shown to be expressed in a number of bovine immune-relevant tissues including CD14^+^ monocytes, mammary epithelial cells, and alveolar macrophages (40–42), however, the genes targeted by the miRNAs in this family are currently unknown.

## Role of the miRNAs in the Bovine Immune System

**Figure 1:**
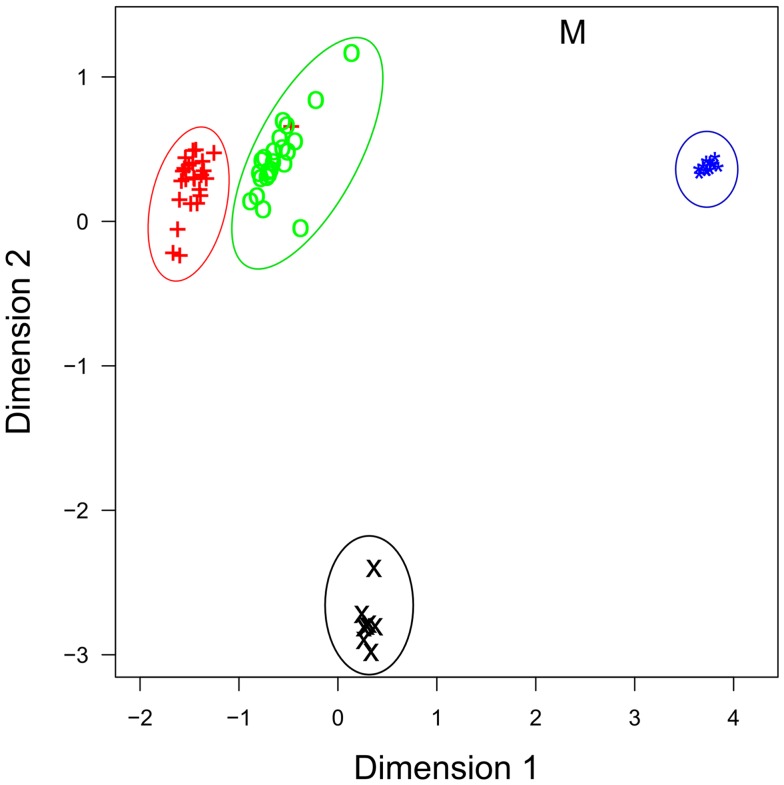
**Multidimensional scaling (MDS) plot of miRNA profiles shows that miRNA expression is cell-type specific**. Similar cell-types have similar profiles, as evidenced by milk (+) and blood CD14^+^ cells (o), and by bovine mammary epithelial cells [primary (*) and cell line (M)], which group together. Data are from four recent RNA-seq publications. +, Blood CD14^+^ monocytes; o, milk CD14^+^ monocytes; ×, bovine alveolar macrophage; *, primary bovine mammary epithelial cells (BME); M, bovine mammary epithelial cell line (MAC-T).

The roles that miRNAs play in regulating immune activation and resolution in response to infection is less well understood in cattle, compared to human and mouse. Investigations in cattle have primarily focused on characterizing miRNA expression in immune-related tissues and investigating whether miRNA expression is perturbed during bacterial/viral infections – but detailed mechanistic studies are, to date, largely lacking. One of the first studies to profile immune-relevant miRNA expression in cattle, characterized the expression of more than 100 bovine orthologs of known human miRNAs, as well as novel bovine miRNAs, in several immune-relevant tissues and provided an early bovine miRNA expression atlas for many later studies (31). More recently, Vegh et al. utilized a next generation sequencing (NGS) strategy to profile miRNA expression on a genome-wide scale in bovine alveolar macrophages, the primary host cell for *M. bovis*, an economically important pathogen (41). NGS has several advantages over classical sequencing technologies, which include the ability to accurately measure expression of all miRNAs simultaneously, with high reliability, single-nucleotide resolution and across the broad dynamic range of expression (43). miRNA expression has now been demonstrated in 10 immune-related bovine tissues (bovine embryo, thymus, small intestine, mesenteric lymph node, abomasum lymph node, CD14^+^ blood isolated monocytes, CD14^+^ milk-isolated monocytes (MIMs), mammary epithelial cells, alveolar macrophages, mammary tissue, and in the MDBK cell line) and tissue-specific expression of miRNAs in these tissues is readily apparent (Figure 1) (31, 40–42, 44–47).

Several studies have also examined whether miRNA expression is altered in response to Gram-positive or Gram-negative infections associated with bovine mastitis, a disease of the bovine mammary gland with significant economic consequences to the dairy industry. Naeem et al. examined a panel of 14 miRNAs from mammary tissue biopsied during an *in vivo* intra-mammary *Streptococcus uberis* infection (46). Four of the fourteen miRNAs were found to undergo differential expression (Table 1). Another study compared transcriptional changes of five inflammation-associated miRNAs, including ones with extensively studied human orthologs: bta-miR-155, bta-miR-146a, and bta-miR-223, in bovine CD14^+^ monocytes stimulated with either LPS or *Staphylococcus aureus* enterotoxin B (SEB) (45). Four miRNAs were differentially expressed, and LPS was found to have a greater effect than SEB at inducing miRNA differential expression.

**Table 1:** **Summary of bovine miRNA literature**.

Reference	miRNA	Tissue	Source	Condition
(31)	Genome-wide	embryo, thymus, lymph node, and small intestine	Holstein–Friesian	None
(44)	Genome-wide	*Bos taurus* kidney cells (MDBK)	Cell line	Bovine herpesvirus 1
(46)	miR-10a, -15b, 16a, -17, -21, 31, -145, 146a, 146b, 155, -181a, -205, -221, and -223	Mammary tissue	Holstein–Friesian	Mastitis
(48)	miR-223	Venous blood	Holstein–Friesian	Mastitis
(45)	miR-9, -125b, -155, -146a, and -223	CD14 monocytes *ex vivo*	Holstein–Friesian	LPS and SEB
(49)	miR-296, -2430, -671, and -2318	Mammary tissue	Holstein–Friesian	Mastitis
(39)	miR-17-5p, -20b, and -93	Mammary tissue	Holstein–Friesian	Mastitis
(40)	Genome-wide	Mammary epithelial cells *ex vivo*	Holstein–Friesian	Mastitis
(41)	Genome-wide	Alveolar macrophages	Holstein–Friesian	None
(39)	Genome-wide	Peripheral blood	Holstein–Friesian	Mastitis
(42)	Genome-wide	CD14 monocytes	Holstein–Friesian	Mastitis
(47)	Genome-wide	MAC-T cells	Cell line	Heat-inactivated *E. coli* or *S. aureus*

More recent studies have employed high-throughput sequencing approaches to temporally profile genome-wide changes in miRNA expression in different cell-types in response to challenge with bovine mastitis-causing pathogens such as *Escherichia coli, S. aureus*, and *S. uberis*. Lawless et al. identified 21 miRNAs that were differentially expressed in bovine mammary epithelial (BMEs) cells challenged *in vitro* with live *S. uberis*, a Gram-positive bacterium (40). Strikingly, the 21 miRNAs differentially expressed in response to this Gram-positive bacterium was substantially different to LPS-responsive miRNAs. Furthermore, the predicted target genes of miRNAs that were down-regulated in BMEs following *S. uberis* infection (but not the targets of up-regulated miRNAs), were statistically enriched for roles in innate immunity, suggesting that the repression of these miRNAs transcriptionally releases the innate immune response to this infection. Subsequently, Jin et al. also reported notable differences in miRNA expression profiles in MAC-T cells challenged with either heat-killed *E. coli* or *S. aureus* (47). This was the first bovine study to directly compare global miRNA expression of two pathogens in the same cell-type.

In the first NGS-based study to temporally profile infection-induced miRNA responses *in vivo*, Lawless et al. simultaneously profiled genome-wide mRNA and miRNA expression at multiple time-points in both milk and blood FACS-isolated CD14^+^ monocytes from cattle infected with *S. uberis* (42). Twenty-six miRNAs and more than 3500 genes were identified as being significantly differentially expressed over the 48 h time-course. In MIMs, up-regulated protein-coding genes were significantly enriched for inflammatory and innate immune pathways, while down-regulated genes were enriched for non-glycolytic metabolic pathways. Monocyte transcriptional changes in the blood were more subtle. Pathway analysis revealed that predicted targets of MIM down-regulated miRNAs were highly enriched for roles in innate immunity, while up-regulated miRNAs preferentially targeted genes involved in metabolism; suggesting that during *S. uberis* infection miRNAs are key amplifiers of monocyte inflammatory response networks and repressors of several metabolic pathways.

To date, only one study has examined whether bovine miRNAs are transcriptionally perturbed during viral infection. In this study, an adult bovine kidney cell line was challenged with *bovine herpes virus 1*. Sequencing 3 miRNA libraries, more than 300 miRNAs were found to expressed, but none were described as being differentially expressed (44).

## Validated Bovine miRNA Target Genes

Two approaches are commonly used to validate miRNA targets. One involves transfection of miRNA mimics (or inhibitors) into cells and confirmation that the expression of predicted target genes is altered as expected (50). This approach provides evidence that a given miRNA can alter the expression of a target gene but does not prove direct regulation (51). The miRNA could, for example, regulate a transcription factor, which subsequently regulates the putative target gene. A more direct approach is to use a reporter assay, where the 3′ UTR of the predicted target gene (or at least the portion containing the predicted miRNA binding site) is cloned upstream of a luciferase or green fluorescent protein (GFP) reporter gene. If binding of the transfected miRNA mimic to the 3′ UTR reduces the level of reporter protein this demonstrates a direct silencing effect of the miRNA on the gene (52).

To date, very few studies have functionally validated bovine miRNA targets. One example where miRNA regulation has been validated is bovine High Mobility Group Box 1 (HMGB1), a nuclear protein, which transcriptionally regulates inflammation (48). Bovine HMGB1 has been shown to be targeted by bta-miR-223, a miRNA that is up-regulated during *S. aureus* infection (48). Similarly, using reporter assays, bta-miR-124 has been shown to regulate expression of Monocyte Chemotactic Protein 1 (MCP1) and Polypyrimidine Tract Binding Protein 1 (PTBP1) in bovine fibroblasts (53).

## Other Areas of Bovine miRNA Immune Biology

In addition to their role as intracellular transcriptional modulators of gene expression miRNAs are also stably expressed in a host of extracellular body fluids including milk, saliva, semen, and plasma (54–57). Extracellular miRNAs can be transferred to distant recipient cells via exosome-mediated transfer and have been demonstrated, in mouse dendritic cells, to modulate recipient cell transcription (58). Exosome packaged miRNAs have been shown to be highly stable and are resistant to degradation by RNases, freeze-thaw, and low pH (56, 59). Exosome miRNAs – including a number of immune-relevant ones, such as bta-miR-223 and bta-miR-125b – have been found in both human breast milk and bovine milk (54–56). MicroRNA expression levels in milk have also been observed to vary during different lactation periods and are present in milk products as well as in raw milk (54). Interestingly, they are particularly abundant in colostrum. Further investigation of the role of exosome packaged miRNAs play in regulating mammalian immunity is urgently needed.

## Future Research Directions in Bovine miRNAs and Their Effect on Immunology, Inflammation, and Infection

It is clear that miRNAs play a key role in regulating human and mouse immune responses. In cattle, studies to date have been mainly limited to demonstrating differential expression of miRNAs in immune-relevant tissues or cells challenged *in vitro* with specific pathogens. Importantly, annotated miRNAs are much fewer in the bovine genome than in murine or human genomes, and a bovine miRNA expression atlas across bovine tissues and cells is needed to bridge this gap. Among its many uses, better annotation of non-coding RNAs would aid in the interpretation of bovine genome-wide association study (GWAS) data. A previously unannotated small non-coding RNA, for example, was recently identified as the only gene in a novel genome-wide significant QTL for somatic cell score, a mastitis indicator trait (60).

Aside from understanding the important basic biology of how miRNAs regulate bovine gene expression, miRNAs could also potentially be of significant utility as biomarkers of specific diseases in cattle. Indeed, miRNAs exhibit many properties that have made them of significant interest as non-invasive biomarkers in human clinical studies. miRNAs are abundantly expressed, in a stable form, in a range of extracellular fluids, are easily measured, and in many cases exhibit temporal and spatial specificity (54–57). They also have high information content – small numbers of miRNAs can serve as accurate biomarkers. Several studies have investigated miRNA expression profiles associated with different mastitis-causing pathogens (Table 1), however, many of these studies were carried out *in vitro*, and the potential of miRNAs as biomarkers of bovine disease is currently limited by a lack of studies of *in vivo* comparison of miRNA profiles associated with multiple different pathogens in the same challenge model. Such *in vivo* studies are needed to identify sensitive and specific biomarkers for particular infections. This could be used for identifying infections, such as tuberculosis, using a simple miRNA-based biomarker, or for distinguishing between different infections, for example between *E. coli* and *S. uberis* driven mastitis, helping veterinarians to select more specific therapeutic strategies.

A further limitation is the fact that research investigating the role of miRNAs in regulating bovine immunity has, to date, focused almost exclusively on bacterial infections and little is known about the role miRNAs play during bovine viral infections. Some of the most economically important and high profile bovine infectious diseases are of viral origin including Foot and Mouth Disease Virus (FMDV) (61), Bovine Viral Diarrheal Virus (BVDV) (62), and the recently described Schmallenberg virus (63). In other species, it is clear that host miRNAs have a direct role in modulating the host immune response to pathogenic viral infections (38). Additionally, some viruses encode their own repertoire of miRNAs to subvert the host immune response. Nearly 200 viral miRNAs have been described (64). Describing their precise effect on the immune response could further our understanding of both bovine miRNA immune biology and virology.

Aside from their utility as biomarkers, miRNAs also have significant potential as therapeutic targets or agents. MicroRNA function can be augmented either by over-expression approaches, using miRNA mimics or vector based over-expression, or by inhibition, using miRNA sponges or anti-miR oligonucleotides (65). In humans, several miRNAs are currently in preclinical and clinical trials as novel therapeutics in cancer, viral infections, and cardiovascular disease (65). Human miR-122, for example, is being investigated for its therapeutic potential to modulate cholesterol metabolism (66). Additionally, targeting miR-122 using the anti-miR miravirsen induces antiviral activity against hepatitis C virus (HCV) (67). The potential clinical utility of miR-122 is being investigated in Phase II clinical trials. Similarly, human miR-208 has been shown to have an important role in modulating cardiac function and remodeling (68), and is currently in preclinical trials. Interestingly from a bovine perspective, this miRNA also has a big impact on metabolism. Treatment with anti-miR-208 prevented weight gain in aging mice, which was due to a reduction in fat weight (69).

All of the miRNAs mentioned above with therapeutic potential have orthologs in cattle and these examples clearly suggest that there is a potential for the application of miRNA-based therapeutic strategies to combat disease and regulate metabolism in cattle in order to influence important economic traits, such as growth, feed efficiency, or milk production. Although the cost of miRNA therapy and the large size of animals may prevent agricultural use, bovine research models still could be valuable, as the conserved nature of miRNAs facilitates translation of research to human application (65).

A current limitation to the translational potential of miRNA biology in cattle is the lack of validated targets for known miRNAs, as only a handful of studies to date have functionally validated predicted miRNA targets (40, 41). Computationally, hundreds if not thousands of putative miRNA targets can be predicted and experimental validation is a costly and labor-intensive procedure. Methods which integrate and correlate miRNA expression with mRNA expression in the same sample can refine computational predictions and increase the validation hit rate. Other more recent technological advancements such as crosslinking immune-precipitation sequencing (CLIP-Seq) can directly identify miRNA targets on a genome-wide scale (70), but such approaches have yet to be implemented in cattle.

In conclusion, miRNAs undoubtedly play a key role in regulating bovine immunity and disease. Future studies are poised to reveal their true potential as novel biomarkers or therapeutic agents in a range of bovine diseases as well as providing further insight into the fundamental biology of how they regulate bovine immune gene expression, insight which is essential before their translational potential can be realized fully.

## Conflict of Interest Statement

The authors declare that the research was conducted in the absence of any commercial or financial relationships that could be construed as a potential conflict of interest.
